# The prominent impairment of liver/intestinal cytochrome P450 and intestinal drug transporters in sepsis-induced acute kidney injury over acute and chronic renal ischemia, a mouse model comparison

**DOI:** 10.1080/0886022X.2019.1602054

**Published:** 2019-04-17

**Authors:** Warumphon Sukkummee, Patcharin Jittisak, Piyanuch Wonganan, Supeecha Wittayalertpanya, Pajaree Chariyavilaskul, Asada Leelahavanichkul

**Affiliations:** a Clinical Pharmacokinetics and Pharmacogenomics Research Unit, Chulalongkorn University, Bangkok, Thailand;; b Department of Pharmacology, Faculty of Medicine, Chulalongkorn University, Bangkok, Thailand;; c Center of Excellence in Immunology and Immune-mediated Diseases, Chulalongkorn University, Bangkok, Thailand;; d Department of Microbiology, Faculty of Medicine, Chulalongkorn University, Bangkok, Thailand

**Keywords:** Cytochrome P450, mouse model, renal impairment, sepsis

## Abstract

Drug dosing adjustment in sepsis-induced acute kidney injury (sepsis-AKI) is currently adjusted based on renal function. Sepsis is a multiorgan injury, and thus, drug metabolism in sepsis-AKI might be interfered by non-renal factors such as changes in functions of drug-metabolizing enzymes in the liver and functions of intestinal drug transporters.

We compared the defect on mouse CYP3A11 (human CYP3A4 representative) in liver and intestine along with several intestinal drug transporters (MDR1a, MRP2, and OATP3) in three mouse models; chronic ischemic reperfusion injury (Chr I/R; 4-week), acute ischemic reperfusion injury (Acute I/R; 24-h), and cecal ligation and puncture (CLP; 24-h) as representative of sepsis-AKI.

Decreased expression of CYP3A11 and drug transporters was demonstrated in all models. Among these models, sepsis-AKI had the least severe renal injury (increased BUN and Scr) with the most severe liver injury (increased ALT and changes in liver histopathology), the most severe intestinal leakage (increased serum (1→3)-β-D-glucan) and the highest increase in serum IL-6. A reduced expression and activity of liver and intestinal CYP3A11 along with intestinal efflux-drug transporter expressions (MDR1a and MRP2), but not drug uptake transporter (OATP3), was predominant in sepsis-AKI compared with acute I/R. Additionally, a reduction of CYP3A4 expression with IL-6 was demonstrated on HepG2 cells implying a direct injury of IL-6 on human liver cells.

Differences in drug metabolism were reported between sepsis-AKI and ischemic-AKI confirming that drug dosing adjustment in sepsis-AKI depends not just only on renal function but also on several non-renal factors. Further studies are warranted.

## Introduction

Chronic kidney disease (CKD) and acute kidney injury (AKI) are worldwide health care problems [1]. Sepsis, ischemia, and nephrotoxicity are the most common cause of AKI [2]. Drug dosing adjustment in CKD and AKI depends only on renal function, represented by glomerular filtration rate (GFR). In fact, overall drug metabolism depends on functions of the liver, kidneys, and gastrointestinal tract. Uremia itself also induces injuries in the liver and gastrointestinal tract as shown by a downregulation of cytochrome P450 (CYP450) enzymes in the liver and intestine and interferes with drug transporter functions [3–5] Indeed, the important role of non-renal clearance in CKD and AKI is reported [3,6].

Sepsis-induced AKI (sepsis-AKI) is a specific category of AKI presented with (1) uremia, (2) tissue ischemia, especially liver and gastrointestinal tract ischemia, and (3) hypercytokinemia [7,8]. The influence of ischemia and/or cytokines against functions of CYP450 has been reported [9,10]. Drug metabolism in sepsis-AKI may be different from ischemic-CKD and ischemic-AKI [11]. It is possible that drug dosing adjustment in sepsis-AKI depends on not only GFR but also the intensity of liver and gut injuries.

Regarding drug metabolism, cytochrome P450-3A (CYP3A) is an important subfamily of CYP450 responsible for the metabolism of more than 50% of drugs [12,13]. As mouse’s CYP3A11 is the best representative of human CYP3A4 compared to other animals, the mouse models were selected to be used in this study [13,14].

In addition, drug transporters including efflux transporters such as P-glycoprotein (P-gp) and multidrug resistance-associated proteins (MRPs), and uptake transporters such as organic anion-transporting polypeptides 3 (OATP3; solute-linked carrier (SLC/SLCO)) also play important roles in drug metabolism [15]. Previous studies in CKD rat models demonstrated a decreased liver and intestinal CYP450 activities, together with a decreased in P-gp activity and changes of other molecules [16–19]. Although the exploration has been done in CKD and AKI, the study in sepsis-AKI and the comparisons between models is still limited.

Here, we explored the influence of renal impairment on drug metabolism represented by changes of CYP3A11 activity and drug transporters in liver and intestine among three mouse models: chronic ischemic reperfusion injury (Chr I/R), acute ischemic reperfusion injury (acute I/R), and sepsis-AKI.

## Materials and methods

### Animals

The animal care and use protocol was approved by the Institutional Animal Care and Use Committee of the Faculty of Medicine, Chulalongkorn University, Bangkok, Thailand, based on the National Institutes of Health, USA’s criteria for the use and treatment of laboratory animals. Eight-week-old male ICR mice were used in this study (National Laboratory Animal Center, Nakhornpathom, Thailand).

### Animal models

Three mouse models of renal injury including Chr I/R (*n* = 12), acute I/R (*n* = 5), and sepsis-AKI (*n* = 5) were studied. Chr I/R model, representative of CKD, was induced according to previous reports [20,21]. In brief, mouse’s left renal artery was clamped for 50 min through left frank incision under ketamine anesthesia on a 37 °C heated operation table. A week later, right nephrectomy was performed under isoflurane anesthesia via a right flank incision. For a sham of Chr I/R model, only renal vessel identification through the left and right frank incisions was performed at one week in parallel with the model. Renal fibrosis was demonstrated after 2–4 weeks post-right nephrectomy in Chr I/R model [20,21].

Acute I/R and sepsis-AKI models were representative of AKI. Acute I/R model was performed with 50-min clamping of bilateral renal arteries through an abdominal incision as previously described [22]. For a sham of Acute I/R model, both renal arteries were identified through abdominal incision before closing the abdominal wall. Sepsis-AKI model was induced by cecal ligation and puncture procedure (CLP) as previously described [23]. In short, after abdominal incision, cecal ligation was performed at 1 cm from the cecal tip and cecum was punctured twice with No.21 needle. For a sham of CLP, cecum was identified through abdominal incision before closing the abdominal wall with similar fluid replacement after an operation. Tramadol (50 mg/kg in 1 mL of 0.9% normal saline) was subcutaneously administered after surgery and at 6-h post-surgery in all procedures for analgesia and fluid replacement.

Chr I/R mice were sacrificed at 2- and 4-week post-nephrectomy with cardiac puncture technique under isoflurane anesthesia. Acute I/R and sepsis-AKI mice were sacrificed at 24-h post-surgery with the same euthanasia method. After sacrifice, serum samples were kept at –80 °C until analysis. Liver and small intestine samples were harvested and snapped frozen into liquid nitrogen and kept at –80 °C until analysis.

### Serum analysis, histopathology, and tissue injury

Renal function was measured as blood urea nitrogen (BUN) and serum creatinine (Scr) using colorimetric assays (QuantiChrom^TM^: DIUR-500 and DICT-500, BioAssay, Hayward, CA, USA). Liver function was measured as serum alanine transaminase (ALT) (EnzyChrom ALT assay, EALT-100, BioAssay). Liver tissue was also fixed and embedded in paraffin and stained with periodic acid–Schiff (PAS) on 4-µm-thickness slides to show liver histopathology. Liver injury was demonstrated by the loss of the pink color in the PAS stain. Serum interleukin-6 (IL-6), a highly reliable proinflammatory biomarker in sepsis [24,25], was measured with an enzyme-linked immunosorbent assay method according to the manufacturer’s instructions (ReproTech, NJ, USA). Small intestinal injury was determined by the translocation of a fungal cell wall molecule (1→3)-β-D-glucan (BG) from intestinal tract into serum using Fungitell assay (Associated of Cape Cod, Falmouth, MA, USA). The increase of serum BG was representative of intestinal injury [8].

### CYP3A activity in liver and small intestine

Liver and small intestine samples were prepared as previously described [26]. In short, all remaining contents in liver and small intestine samples were removed by pressing down with wet fingers and gently rinsed with ice-cold 0.9% normal saline. The mucosal surface of the luminal wall was scrapped and placed in 5 mL of homogenizing buffer in borosilicate homogenization tube. The samples then underwent homogenized afterward. The homogenate was centrifuged at 9200 rpm at 4˚C for 20 min. The supernatant was collected and further centrifuged at 95,000 rpm at 4˚C for 20 min. The pellets were resuspended in 0.5 mL of homogenizing buffer. Microsomal protein concentrations were then determined (Bio-Rad Dc Protein Assay, BIO-RAD Laboratories Life Science Group, CA, USA).

For the activity of CYP3A in microsomal liver and small intestine, 1000 µg/mL of microsomal protein was added with 0.2 M of potassium phosphate buffer and 100 mM of MgCl_2_ [26]. Midazolam (100 µM) and testosterone (34.67 mM) as substrates for enzymatic reactions were incubated with microsomes at 37˚C for 5 min. Then, 5 µL of 1 unit/µL glucose-6-phosphate dehydrogenase enzyme was added and incubated at 37˚C for 1 h. At the end of 1-h incubation, 5 mL of dichloromethane was added to stop the reactions. The organic phase was evaporated at 30 °C until dry and further reconstituted with 50 µL of mobile phase.

The peak areas of the metabolites of midazolam and testosterone (1’-hydroxymidazolam and 6ß-OH-testosterone, respectively) representing CYP3A activities were measured by a validated high-performance liquid chromatography (HPLC) technique as previously described with modifications [26]. In brief, 30 µL of reconstituted sample was injected into the HPLC system (Shimadzu LC-20A HPLC system). Diazepam (100 µg/mL) and 11-alpha-hydroxyprogesterone (80 µg/mL) were used as internal standards for midazolam and testosterone, respectively. The analytes were separated on HPLC column (C18, particle size 3.5 μm, dimensions 4.6 × 100mm, Waters). 10 µL of 1’-hydroxymidazolam and 10 µL of diazepam were isocratically eluted with 10 mM sodium acetate buffer (pH 4.7): acetonitrile (55:45) as mobile phase. The flow rate was 0.8 mL/min at 30^º^C. The absorbance of the eluents was 220 nm. Ten microliters of 6ß-OH-testosterone and 10 µL of 11-alpha-hydroxyprogesterone were isocratically eluted with H_2_O: methanol (50:50) as mobile phase. The flow rate was 0.7 mL/min at 40^º^C. The absorbance of the eluents was 280 nm. The coefficient of variations of each test was less than 10% at each concentration studied. The activity of the enzyme was finally calculated and expressed as units/mg protein.

### mRNA and protein expressions of drug transporters and CYP3A11 in liver and small intestine

At the time of sacrifice, liver and duodenum (defined as the segment of small intestine within 3-cm distal from pyloric sphincter of stomach) samples were rinsed with ice-cold 0.9% normal saline before snapped frozen into liquid nitrogen and kept at –80^º^C until RNA isolation. Total RNA was extracted from frozen tissue using TRIzol reagent (Invitrogen, Burlington, ON, Canada) according to manufacturer’s instructions. One microgram of total RNA was used to prepare cDNA using ImProm-II Reverse Transcription System (Promega). The mRNA encoding for P-gp (MDR1a), MRP2, OATP3, and CYP3A11 was measured by TaqMan gene expression assays (Applied Biosystems, Carlsbad, CA). Results were reported as relative quantitation using comparative threshold (delta-delta Ct) method (2^–ΔΔCt^) as previously reported [27]. The amount of the target gene in the sample was normalized to glyceraldehyde-3-phosphate dehydrogenase as an endogenous housekeeping gene.

Protein expression of CYP3A11 and drug transporters including MDR1 and MRP2 was assessed on liver microsomal fraction using Western blot analysis. In short, 60 µg of microsomal protein was electrophoresed on a 7.5% polyacrylamide gel containing 0.1% SDS. Proteins were transferred to nitrocellulose, and immunoblots were performed according to antibody’s manufacturer’s recommendation. Immune complexes were revealed by horseradish peroxidase (HRP) (Millipore, Bllerica, MA). Band intensity was determined by densitometry (Quantity One 1-D Analysis software on VersaDoc Imaging System, Bio-Rad Laboratories). Primary antibody for CYP3A11 and MDR1 was obtained from Santa Cruz Biotechnology (Santa Cruz, CA), and MRP2 was from Abcam (Cambridge, UK). Actin was obtained from Cell Signaling Technology (Beverly, MA). HRP-linked secondary antibodies were obtained from Cell Signaling Technology (Beverly, MA). Luminata Forte HRP substrate was obtained from Merck.

### In vitro study: the influences of IL-6 on hepatocytes

A cell line of human hepatoma (HepG2 cells) was cultured in Dulbecco's modified eagle medium supplemented with 10% fetal bovine serum and 1% penicillin streptomycin. 1 × 10^5^ cells of HepG2 cells were seeded into a 24-well plate for 24 h. To explore the influence of IL-6 and urea on hepatocyte functions, the cells were incubated with human IL-6 (R&D Systems, Minneapolis, MN, USA) and urea (Sigma-Aldrich, St. Louis, MI, USA) at for 24 h at 50 and 100 ng/mL of IL-6. The total RNA was extracted by RNA easy mini kit (Qiagen, Hilden, Germany). One microgram of total RNA was processed for the reverse transcription with a high**-**capacity reverse transcription assay (Applied Biosystems, Warrington, UK). Real**-**time polymerase chain reaction (PCR) was performed on Applied Biosystems 7500 Real-Time PCR System using SYBR® Green PCR Master Mix (Applied Biosystems, Warrington, UK). The primer for CYP3A4, a resemblance human gene with mouse CYP3A11, was CYP3A4-F_5’-CAC AGA TCC CCC TGA AAT TAC GCT TA-3’, CYP3A4-R_5’-AAA ATT CAG GCT CCA CTT ACG GTG-3’. Results were reported as relative quantitation using comparative threshold (delta-delta Ct) method (2^–ΔΔCt^) as previously described [27]. The amount of the target gene in the sample was normalized to β-actin as an endogenous housekeeping gene.

### Statistical analysis

Continuous data were reported as mean and standard error of the mean (mean ± SE) unless otherwise stated. The differences between two groups were examined by the Student’s *t-*test, and the differences between more than two groups were examined by one-way analysis of variance (ANOVA) followed by Tukey’s analysis. All statistical analyses were performed with SPSS 11.5 (SPSS, IL, USA). A *p* value <0.05 was considered statistically significant.

## Results

Because of the similarity between mouse-CYP3A11 and human-CYP3A4, CYP3A11 expression and activity were explored with some intestinal drug transporters in mouse models of I/R (acute and chronic) and sepsis.

### Effects of chronic ischemic reperfusion injury on liver and intestinal CYP3A11 and intestinal drug transporters

**Figure 1.**
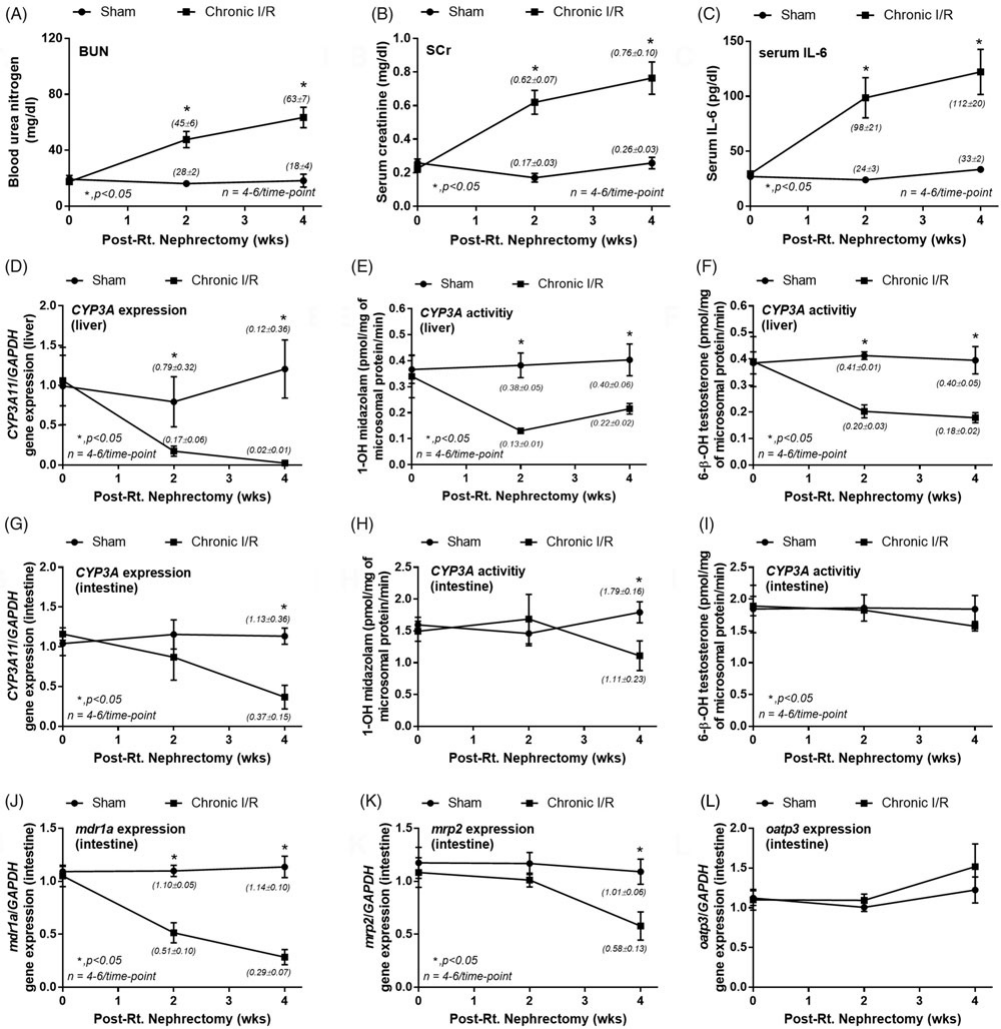
Characteristics of chronic ischemic reperfusion injury (chronic I/R) and sham control by blood urea nitrogen (BUN; A), serum creatinine (Scr; B) and serum interleukin-6 (IL-6; C). The alteration in liver (D–F) and intestinal (G–I) cytochrome P3A11 (CYP3A11) expression and activity using midazolam or testosterone as substrates together with intestinal drug transporters expression (MDR1a, MRP2, and OATP3; J–L) were also demonstrated. The parameters were measured at 0, 2 and 4 weeks post-right nephrectomy, and the data from normal mice were used for 0-week time point.

Chr I/R model represents the influence of chronic uremia on drug metabolism [20,21]. Figures 1A–C show increased BUN, Scr, and IL-6 in Chr I/R mice at 2- and 4-week post-nephrectomy confirming an impairment of renal function compared with controls.

The reduction of liver CYP3A11 mRNA expression (Figure 1D) and CYP3A11 activity by midazolam (Figure 1E) and testosterone (Figure 1F) as substrates were shown. In parallel, a decreased intestinal CYP3A11 mRNA (Figure 1G) and CYP3A11 activity by midazolam (Figure 1H), but not testosterone (Figure 1I), was demonstrated at week 4.

In regard to intestinal drug transporters, mRNA expressions of MDR1a decreased as early as week 2 and worsen at week 4 (Figure 1J). MRP2 mRNA expression was significantly lower than the control group only at week 4 (Figure 1K). In contrast, OATP3 expressions, which, at least in part, responsible for the control of uremic toxins [28], were not different between the groups (Figure 1L).

### Effects of acute ischemic reperfusion injury and sepsis on liver and intestinal CYP3A11 and intestinal drug transporters

While the pathophysiology of sepsis is the combination of multi-organ ischemia and hyper-cytokinemia [29–31], the pathophysiology of chronic and acute I/R is mainly related to renal ischemia.

**Figure 2.**
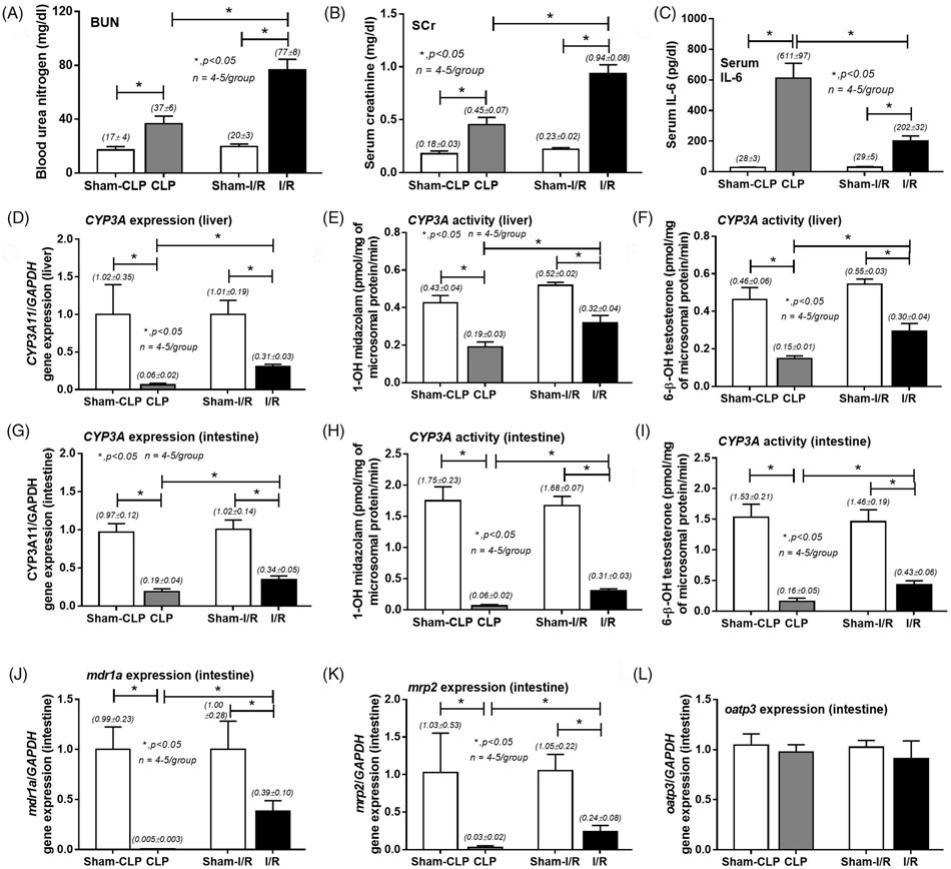
Characteristics of cecal ligation and puncture induced sepsis (CLP) an acute ischemic reperfusion injury (I/R) and sham control at 24-h post-operation by blood urea nitrogen (BUN; A), serum creatinine (Scr; B) and serum interleukin-6 (IL-6; C). The alteration in liver (D–F) and intestinal (G–I) cytochrome P3A11 (CYP3A11) expression and activity by midazolam or testosterone as substrate together with intestinal drug transporters expression (MDR1a, MRP2, and OATP3; J–L) were also demonstrated.

Interestingly, a model of acute I/R showed higher BUN and Scr at 24 h, but lower serum IL-6 in comparison with sepsis-AKI model (Figures 2A–C). Liver and intestinal CYP3A11 mRNA expressions (Figures 2D and G) and CYP3A11 activities (Figures 2E–F and 2H–I) as well as intestinal MDR1a and MRP2 mRNA expressions in both acute I/R and sepsis-AKI models were lower than controls (Figures 2J and K). All reductions were more predominant in sepsis-AKI model. Consistent with results found in Chr I/R model, OATP3 mRNA expressions were not affected (Figure 2L).

### Comparisons of three models

**Figure 3.**
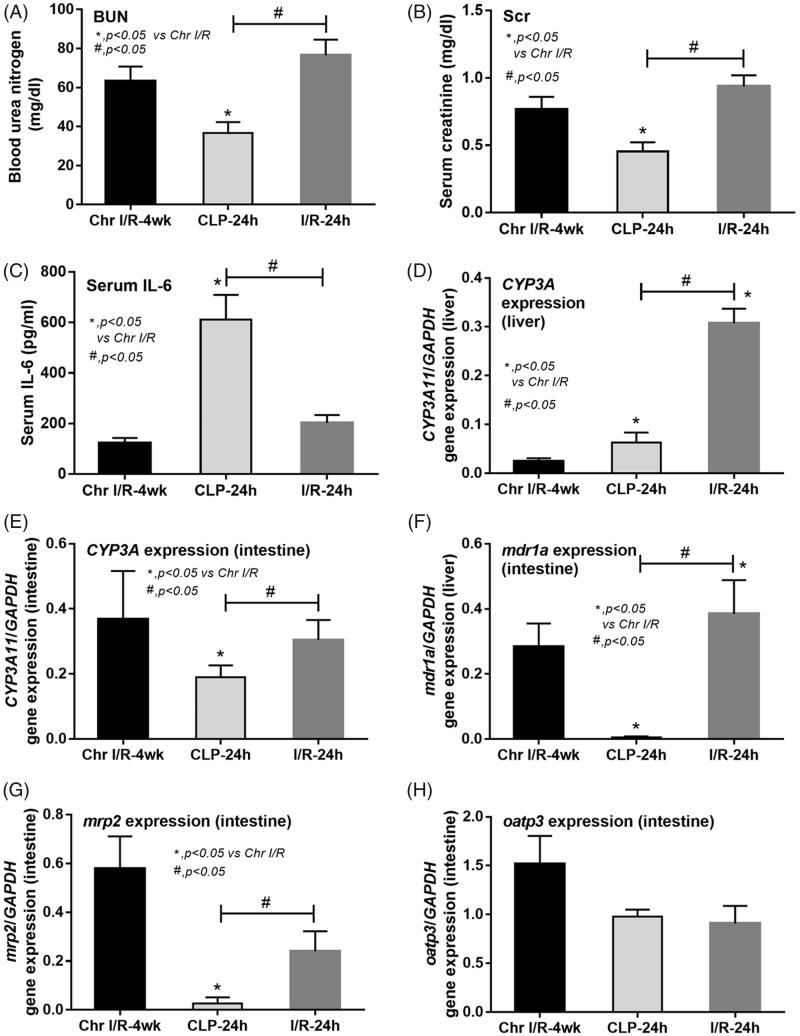
Comparisons of chronic ischemic reperfusion injury at 4 weeks post-right nephrectomy (Chr I/R-4 weeks), 24-h post-cecal ligation and puncture sepsis (CLP-24 h) and 24 h after acute ischemic reperfusion injury (I/R-24 h) in terms of renal injury parameters including blood urea nitrogen (BUN; A), serum creatinine (Scr; B), and serum interleukin-6 (IL-6; C). The alteration in liver (D) and intestinal (G) cytochrome P3A11 (CYP3A11) expression together with intestinal drug transporters expression (MDR1a, MRP2, and OATP3; F–H) was also demonstrated.

The comparisons of the three models were performed between Chr I/R model at week 4, acute I/R model at 24 h and sepsis-AKI model at 24 h (Figures 3A–H) due to the similarity in severity of renal injury, presented by BUN and Scr, of Chr I/R models at week 4, acute I/R models at 24 h, and the rapid disease progression in sepsis-AKI models in which mice died as early as 24 h.

**Figure 4.**
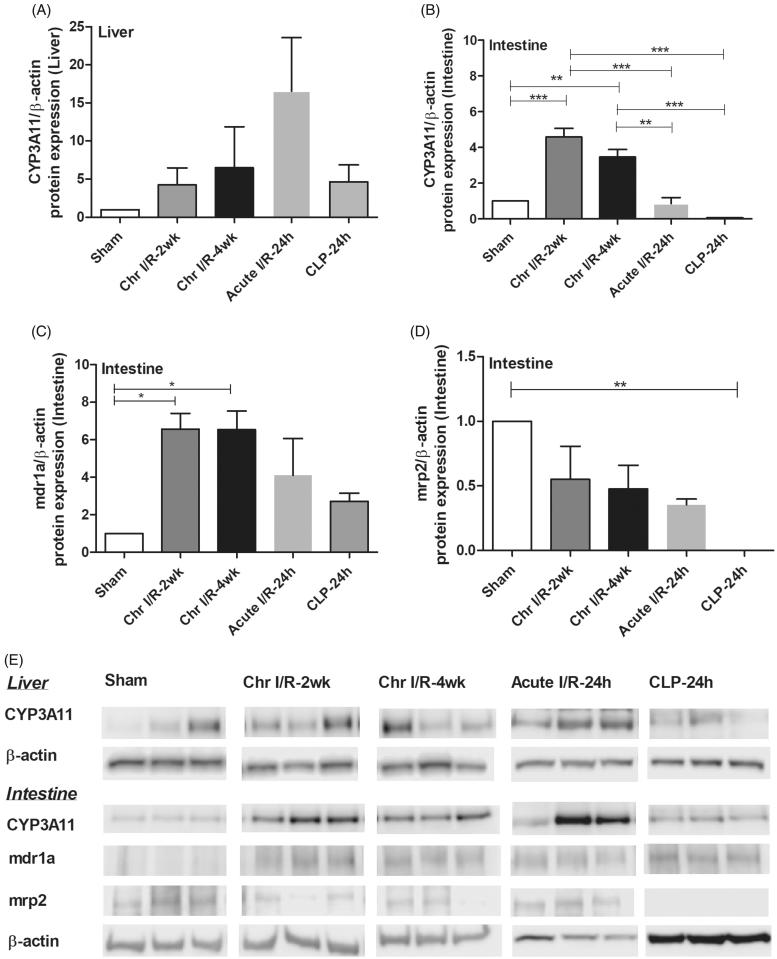
Comparisons of protein expression in chronic ischemic reperfusion injury at 4 weeks post-right nephrectomy (Chr I/R-2 weeks and Chr I/R-4 weeks), 24-h post-cecal ligation and puncture sepsis (CLP-24 h), and 24 h after acute ischemic reperfusion injury (I/R-24 h). Data were liver CYP3A11 protein expression (A), intestinal CYP3A11 protein expression (B), intestinal MDR1 protein expression (C) and intestinal MRP2 protein expression (D). Representative blots are shown in (E).

Despite a subtle increase in Scr, serum IL-6 levels in sepsis-AKI models were 6–7 times higher than the levels in both I/R models (Figures 3A–C). Interestingly, the liver CYP3A11 mRNA expression was lower in Chr I/R model and sepsis-AKI model than acute I/R model, demonstrating the predominant influence of chronic uremia over acute uremia and sepsis (Figure 3D). This was confirmed by protein expression (Figure 4A) mRNA. Expressions of intestinal CYP3A11 and drug transporters including MDR1a and MRP2, but not OATP3, were decreased in sepsis-AKI (Figures 3E–H). Protein expression showed that CYP3A11 and MRP2 markedly decreased in sepsis (Figures 4B and D). Although MDR1 protein expression was not significantly reduced compared to control, the expression in sepsis was lower than that of ischemic models (Figure 4C).

### Confirmation of the effects of ischemia and IL-6 in drug metabolisms

**Figure 5.**
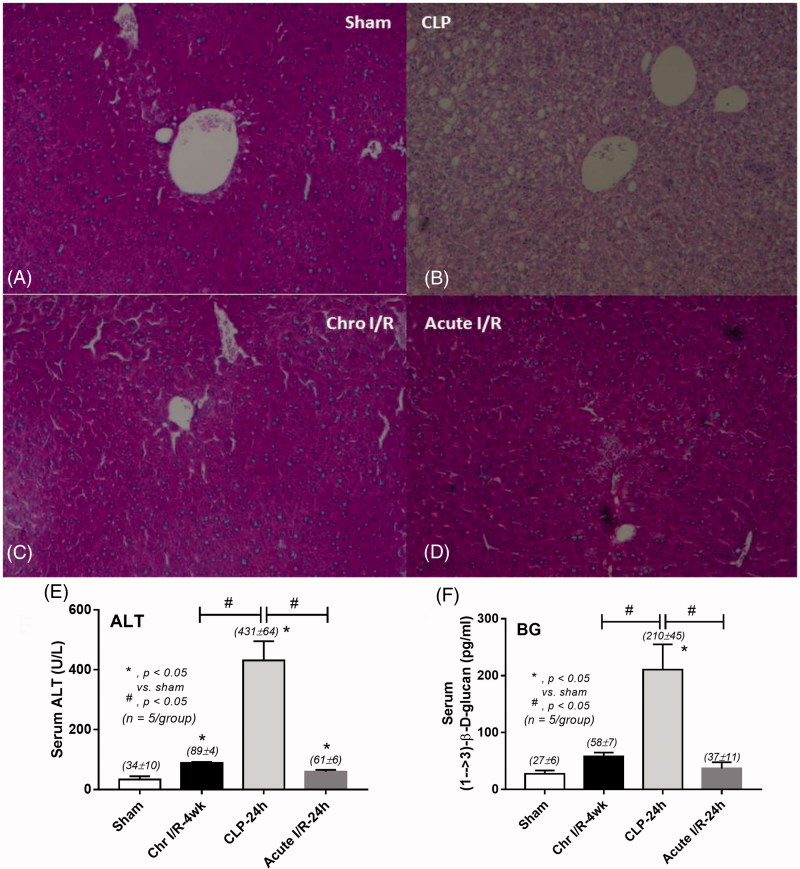
The liver injury was demonstrated by changes in liver histopathology in 24-h post-cecal ligation and puncture sepsis (CLP), chronic ischemic reperfusion injury (Chr I/R) at 4 weeks post-right nephrectomy, and 24 h after acute ischemic reperfusion injury (acute I/R) (A–D) and serum alanine transaminase (ALT) concentrations (E). The intestinal injury was demonstrated by serum (1→3)-β-D-glucan (BG; F).

The loss of glycogen storage [32] as shown by reduced PAS staining color (Figures 5A–D) and an increase in serum ALT (Figure 5E) are signs of severe liver injury in sepsis seen in this study. These changes might be responsible for a reduction of liver CYP3A11 activity. Severe sepsis induces intestinal ischemia as determined by an increase in serum BG (Figure 5F), an indirect indicator of the loss of intestinal permeability [8], and might be associated with decreased drug transporters expressions (Figures 3F–G).

**Figure 6.**
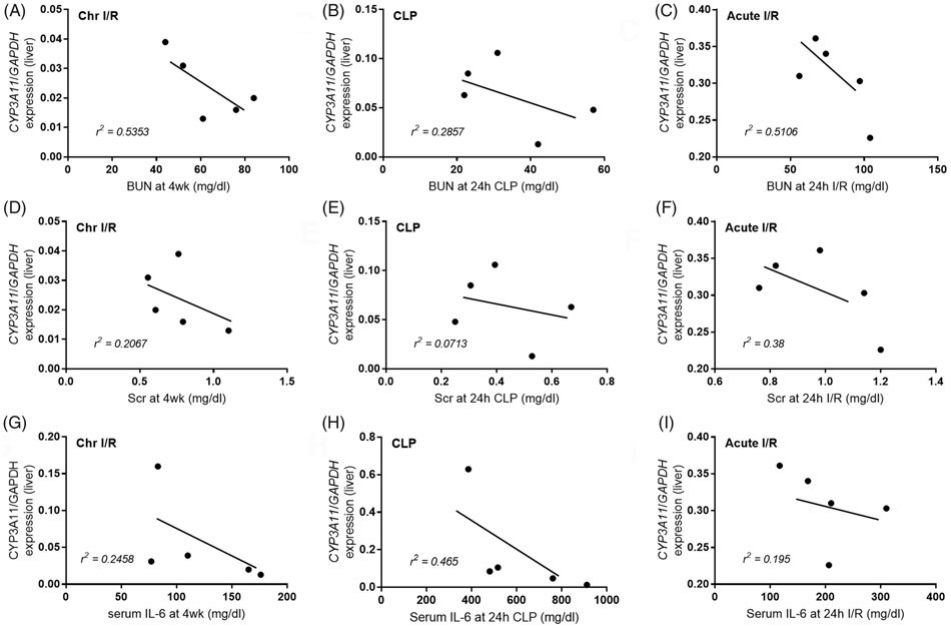
The correlations between CYP3A11 expression and blood urea nitrogen (BUN; A–C), serum creatinine (Scr; D–F) and serum interleukin-6 (IL-6; G–I) were demonstrated. Models are chronic ischemic reperfusion injury (Chr I/R), cecal ligation and puncture-induced sepsis (CLP) an acute ischemic reperfusion injury (acute I/R).

Additionally, in sepsis, high negative correlations between liver CYP3A11 expression and serum IL-6, but not BUN, were demonstrated (Figure 6H, *r^2^*=0.4650). In contrast, BUN was associated with decreased CYP3A11 expression in both chronic and acute renal ischemic models (Figure 6A, *r^2^*=0.5353 and Figure 6C, *r^2^*=0.5106, respectively), but less association was found sepsis-AKI model (Figure 6B, *r^2^*=0.2857). These suggest the different effects of uremia and serum IL-6 on CYP450 enzyme [33,34].

**Figure 7.**
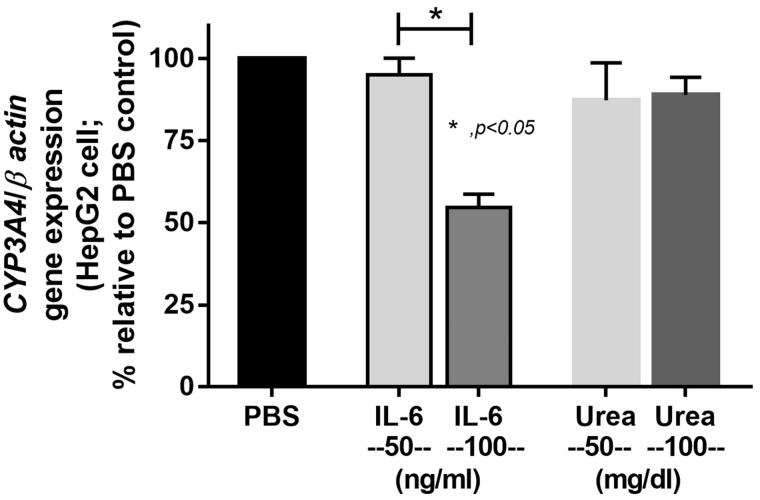
The activation of CYP3A4 expression on a hepatoma cell line (HepG2 cells) with interleukin-6 (IL-6) and urea was demonstrated.

The influences of IL-6 *versus* urea on CYP3A4 in human hepatocyte cell lines were also explored. Although there were limitations of drug transporter studies using intestinal cell lines [35,36], HepG2 cells are acceptable representative of human liver cell to assess CYP450 function [37]. Indeed, IL-6 at the concentration of 100 ng/mL independently reduced the expression of CYP3A4 on HepG2 cells (Figure 7).

## Discussion

On the basis that drug metabolism associates with functions of several organs including liver, kidneys and gastrointestinal tract, nonetheless, the drug dosing adjustments in CKD mainly depends on GFR. Despite dosage adjustments, CKD patients still suffer from a great number of unwanted drug adverse events. Oral bioavailability of drugs depends on many factors such as intestinal drug transporters and activity of liver metabolizing enzymes [38]. Liver injury associated with renal impairment was also demonstrated in previous reports [3–5]. Sepsis is a multi-organ injury that could affect all important organs involved in the process of drug metabolism [39]. Hence, it is possible that dose adjustment of several drugs in sepsis, especially sepsis-AKI, might be different from other types of AKI.

This study explored the effects of renal impairment on drug metabolism represented by changes of CYP3A11 activity and drug transporters in the liver and intestine among three mouse models: Chr I/R, acute I/R and sepsis-AKI models. Even though the severity of renal impairment in sepsis was lower compared with ischemic models, we found a greater reduction of CYP3A11 activity and expression of efflux drug transporters on sepsis-AKI model. These data confirm that drug dosing adjustment in sepsis-AKI depends not just only on renal function but also on several non-renal factors.

### A prominent suppression of liver CYP3A11 in chronic ischemia and sepsis-AKI

Liver CYP3A11 expression and activity were lower than control in all models with greater suppressions seen in chronic ischemia and sepsis than acute ischemia. Among the three mouse models, liver injury was most severe in sepsis as demonstrated by the loss of glycogen storage and increased serum ALT. Indeed, uremia-induced liver injury was shown in previous studies in chronic and acute renal ischemia [7,40] and also presented here as the subtle elevation of serum ALT in ischemic models. Therefore, it is not surprising that liver CYP3A11 expression and activity were very low in sepsis.

High serum IL-6 in sepsis might also influence an expression of CYP3A11 as demonstrated by the correlation between CYP3A11 and serum IL-6 in sepsis-AKI model together with *in vitro* IL-6 enhanced CYP3A11 expression on HepG2 cells.

However, CYP3A11 levels in sepsis were similar to those in chronic ischemic model implying the potent effect of chronic uremia exposure. The influence of chronic uremia against CYP3A11 was also demonstrated by the correlation between CYP3A11 expression and BUN in Chr I/R model. In contrast, CYP3A11 defect in acute I/R models was not as severe as in Chr I/R models supporting a time-dependent characteristic of uremic-induced acute liver injury.

Although we did not have *in vivo* data on drug metabolism, CYP3A11 activity was presented in *ex vivo* analysis by midazolam and testosterone as reagents with CYP3A11-dependent metabolism.

### A prominent suppression of intestinal CYP3A11 in sepsis-AKI

Unlike the prominent liver CYP3A11 suppression seen in CKD, the intestinal CYP3A11 was dominantly reduced in sepsis-AKI model. In line with this, intestinal injury as demonstrated by the defect of intestinal permeability (spontaneous elevation of serum BG) was most severe in sepsis model. Hence, the suppression of intestinal CYP3A11 was, at least in part, due to the poor tissue perfusion of the intestinal system during sepsis.

However, there might be a drug-dependent effect on the activity of intestinal CYP3A11. The intestinal CYP3A11 expression was low in chronic ischemic renal impairment at week 4, but a reduction in CYP3A11 activity was found only when midazolam was introduced as a substrate. Thus, the expression might not be associated with the enzyme activity in some drugs. However, the CYP3A11 expression and activity were concordant in most of other tests. Perhaps, some unknown factors of chronic ischemic model would play a role. More studies are needed to clarify this issue.

### A prominent suppression of intestinal drug transporters in sepsis-AKI

As bowel ischemia and intestinal leakage are the prominent characteristics in sepsis-AKI, the defect in intestinal drug transporters in sepsis is possible. The expression of efflux transporters (MDR1a and MRP2) and uptake transporters (OATP3) which are responsible for drug efflux into intestinal tract and drug reabsorption into blood circulation, respectively, was assessed.

Interestingly, the efflux transporters (MDR1a and MRP2) were very sensitive to uremia as a marked reduction in expression was found in both acute and chronic I/R. A more severe reduction in transporter expression was shown in sepsis-AKI. Therefore, drugs that are excreted mainly via efflux transporters such as digoxin, fexofenadine, sulfate-conjugates, and cyclosporine might be easier to reach toxic levels in patients with sepsis-AKI whose drug dose is commonly adjusted only with renal function. More studies in this topic are required [41].

In contrast, the uptake transporter (OATP3) was protected from uremia and ischemia. Although there was less report on a safeguard of OATP3 during sepsis, several previous publications demonstrated a uremic resistance of OATP3 [28,41,42]. In addition, OATP3 is also responsible for the intestinal uptake of intestinal-derived uremic toxin, for example indoxyl sulfate, to enhance the translocation of toxins from gut to serum [43]. Levels of drugs and xenobiotics that are reabsorbed through OATP3 such as bile acids, bilirubin, eicosanoids, steroid conjugates, and thyroid hormone might be enhanced in kidney injury and sepsis [16].

CYP450 regulation in CKD could be altered by several mechanisms. The accumulation of uremic toxins includes macromolecules such as parathyroid hormone and inflammatory cytokines in blood which interfere with intracellular signaling pathways leading to blockade of CYP gene transcription and resulting in a reduction of CYP mRNA production and further interferes with CYP functions. The other mechanism is a direct inhibition of CYP metabolism by uremic toxins as they enter into the cell. This mechanism is supported by the evidence that hemodialysis temporary restores normal CYP metabolism. Additionally, epigenetic mechanisms may play a role in CYP regulation in CKD [44]. Mechanisms of AKI cause liver dysfunction was reported as leukocyte infiltration, increased oxidative stress and liver cell apoptosis. These lead to altered CYP450 enzyme activity [31,39]. Results of this study support those proposed mechanisms where accumulation of uremic toxins altered CYP3A11 expression and activity and this was time-dependent, whereas the mechanism of changes in CYP expression and activity in sepsis-AKI was more associated with inflammation evidenced by marked increased in IL-6 levels.

The limitation of this study was that the ischemic reperfusion injury and sepsis models were not totally comparable. However, we carefully chose to compare the relatively comparable severity of renal impairment of all groups to make sure that the results presented in sepsis group were responsible by non-renal factors as much as possible. Of note, sepsis-AKI is a multi-organ injury disease that leads to several other organ dysfunctions [39]. As we focused mainly on liver dysfunction, the mechanism proposed for liver dysfunction is inflammation [44]; therefore, we measured a biomarker of proinflammatory process (IL-6).

In conclusion, although serum urea and creatinine in sepsis were lower than ischemic models, the severity of a reduction in CYP3A11 expression and activity and a reduction in efflux drug transporters expression were higher. Sepsis-induced poor tissue perfusion and inflammation might be the main factors drive all these abnormalities.

Serum IL-6 was also prominent in sepsis than ischemic models. While the renal excretory defect led to in an increase in serum IL-6 in acute and chronic ischemic AKI models, serum IL-6 in sepsis is predominantly increased from the inflammation-enhanced production [24,25] and might also directly interfere with liver CYP3A11 production.

These data support the different effects on drug metabolism between sepsis-AKI and ischemic renal impairment. Health care providers should consider drug dosing adjustment based on renal and non-renal including liver drug-metabolizing enzymes and intestinal drug transporters functions in patients with sepsis as a proper strategy for this specific group of patients. More studies are warranted.
